# The risk of various types of cardiovascular diseases in mutation positive familial hypercholesterolemia; a review

**DOI:** 10.3389/fgene.2022.1072108

**Published:** 2022-12-06

**Authors:** Anders Hovland, Liv J. Mundal, Marit B. Veierød, Kirsten B. Holven, Martin Prøven Bogsrud, Grethe S. Tell, Trond P. Leren, Kjetil Retterstøl

**Affiliations:** ^1^ Nordland Heart Center, Bodø, Norway; ^2^ The Lipid Clinic, Oslo University Hospital, Oslo, Norway; ^3^ Oslo Centre for Biostatistics and Epidemiology, Department of Biostatistics, University of Oslo, Oslo, Norway; ^4^ Department of Nutrition, University of Oslo, Oslo, Norway; ^5^ National Advisory Unit on Familial Hypercholesterolemia, Oslo University Hospital, Oslo, Norway; ^6^ Unit for Cardiac and Cardiovascular Genetics, Oslo University Hospital, Oslo, Norway; ^7^ Department of Global Public Health and Primary Care, University of Bergen, Bergen, Norway; ^8^ Division of Mental, Bergen, Norway

**Keywords:** familial hypecholesterolemia, ASCVD, LDL, cholesterol, statin (HMG-CoA reductase inhibitor), aortic valve stenosis, stroke, myocardial infaction

## Abstract

Familial hypercholesterolemia (FH) is a common, inherited disease characterized by high levels of low-density lipoprotein Cholesterol (LDL-C) from birth. Any diseases associated with increased LDL-C levels including atherosclerotic cardiovascular diseases (ASCVDs) would be expected to be overrepresented among FH patients. There are several clinical scoring systems aiming to diagnose FH, however; most individuals who meet the clinical criteria for a FH diagnosis do not have a mutation causing FH. In this review, we aim to summarize the literature on the risk for the various forms of ASCVD in subjects with a proven FH-mutation (FH+). We searched for studies on FH+ and cardiovascular diseases and also included our and other groups published papers on FH + on a wide range of cardiovascular and other diseases of the heart and vessels. FH + patients are at a markedly increased risk of a broad range of ASCVD. Acute myocardial infarction (AMI) is the most common in absolute numbers, but also aortic valve stenosis is by far associated with the highest excess risk. Per thousand patients, we observed 3.6 incident AMI per year compared to 1.9 incident aortic valve stenosis, however, standardized incidence ratio (SIR) for incident AMI was 2.3 compared to 7.9 for incident aortic valve stenosis. Further, occurrence of ischemic stroke seems not to be associated with increased risk in FH+. Clinicians should be aware of the excess risk of almost all kind of ASCVD in FH+, and the neutral risk of stroke need to be studied further in FH + patients.

## Introduction

Atherosclerotic cardiovascular diseases (ASCVDs) comprising coronary heart disease, cerebrovascular disease, peripheral artery disease and aortic disease are common diseases affecting a huge number of patients worldwide (Virani et al., 2021). Low-density lipoprotein cholesterol (LDL-C) is an important risk factor for ASCVD, and familial hypercholesterolemia (FH) is a common, inherited disease (Hu et al., 2020) characterized by high levels of LDL-C from birth. FH could be considered a model disease for studying the pathophysiological effect of LDL-C (Domanski et al., 2020). Therefore, any diseases associated with increased LDL-C levels would be expected to be overrepresented among FH patients.

A recent editorial by Khera and Hegele asked the seemingly unnecessary question—“What Is Familial Hypercholesterolemia, and Why Does It Matter?”. Different definitions of FH lead to different groups of patients being identified as having FH (Khera and Hegele, 2020).There are several clinical scoring systems aiming to diagnose FH including the MedPed Criteria (MedPedProgram, 2005), the Simon Broome Register criteria (Group, 1991) and the modified Dutch Lipid Clinic Network score (Betteridge, 2000). These scoring algorithms for FH are useful for identifying individuals at high risk of ASCVD, but it has become evident that most individuals who meet the clinical criteria for probable or definite FH diagnosis do not have a mutation causing FH (Lee et al., 2019). However, those with a pathogenic FH mutation causing high LDL-C levels align with the classical concept of FH as a monogenic autosomal genetic disease (Khera and Hegele, 2020). Trinder et al. have clearly shown that mutation-positive FH patients, have increased risk of cardiovascular disease when compared to those with polygenic hypercholesterolemia (Trinder et al., 2019). Different criteria for FH create a knowledge gap for uniform risk assessment. Our aim with this article is to describe the risks associated with patients with a confirmed genetically verified pathological FH mutation.

In this review, we aim to summarize the literature on the risk for the various forms of ASCVD in subjects with a genetically proven FH-mutation (FH+).

In the present European guidelines for management of dyslipidemias, FH individuals are categorized as individuals at high-risk of disease by default, and in primary prevention treatment goals are at least a 50% reduction from baseline in LDL-C down to <1.8 mmol/L or <1.4 mmol/L depending on the global risk burden (Mach et al., 2019). However, very few patients with FH + reach treatment target even on maximal treatment with statins and ezetimibe and the risk of ASCVD remain high in FH + compared to controls (Arnesen et al., 2020). Use of PCSK9-inhibitors in addition to statins and ezetimibe can bring LDL-C down in many FH + patients, but its use is limited by high cost (Attipoe-Dorcoo et al., 2021). Also newer treatment options have been introduced including evinacumab, a monoclonal antibody inhibiting the angiopoetin-like 3, a hormone regulating the lipoprotein lipase (Raal et al., 2020).

Then we also included our and other groups published papers on FH + on a wide range of cardiovascular and other diseases of the heart and vessels including; coronary heart disease, cerebrovascular disease, peripheral artery disease, aortic disease, ASCVD mortality, heart failure and atrial fibrillation and aortic valve stenosis.

## Materials and methods

We searched PubMed using the search string “[(familial hypercholesterolemia AND cvd AND mutation)” or “(familial hypercholesterolemia AND cvd AND genotyped)”]. No limitation for search period was used. Date of search was 1 July 2021. We identified 132 papers that were reviewed by two authors (KR and AH) to identify the inclusion criteria which were 1) a confirmed pathogenic FH mutation in more than 90% of the study population, 2) heterozygous FH only, not homozygous, 3) CVD outcomes clearly reported, 4) English language, 5) studies reporting on quantitative data, not qualitative studies, and not single case reports. Review articles and letters to the editor were also excluded. Nine articles on CVD in mutation positive FH were included and discussed below. The references of these articles were then reviewed to identify additional studies and we also performed hand searches of papers in our collections and the reference lists of extensive review articles.

Then we also included our and other groups’ published papers on FH + on a wide range of cardiovascular and other diseases of the heart and vessels including; coronary heart disease, cerebrovascular disease, peripheral artery disease, aortic disease, ASCVD mortality, heart failure and atrial fibrillation and aortic valve stenosis. Lastly, we explore the spectrum of ASCVD in mutations positive FH patients, utilizing data from the Norwegian Register of FH+. In 2021, this register reported to have identified 264 different mutations to cause FH, of which 94.0% is in the *LDL-receptor* gene. Two mutations in the *APOB* gene (5.4%) and three mutations in the *PCSK9* gene (0.6%) have been found to cause FH.

## Familial hypercholesterolemia-mutation and cardiovascular disease in general

(Table 1) Receptor negative FH, in which there are no detectable LDL-receptor activity, was compared to receptor defective mutations, in which there are some LDL-receptor activity, and no difference in atherosclerotic disease assessed by measurement of intima-media thickness with carotid ultrasound and coronary calcification by CT spiral scan was found (Brorholt-Petersen et al., 2002).

**TABLE 1:** CVD in mutation FH + patients.

Group	N =	Main finding
Brorholt-Petersen	62	LDL receptor activity did not affect IMT or coronary calcium
Umans-Eckenhausen	608	LDL receptor type only partially contribute to future CVD risk
Koeijvoets	593	LDL receptor type in children affects CVD risk in the parents
Civeira	951	Tendon xanthomas were associated with CVD risk
Soška	555	LDL particle size was associated with CVD risk
Besseling	14 283	LDL-C levels >8.0 mmol/L increased the risk of CVD
Paquette	725	Genetic risk scores further predict CVD risk in FH patients
Fantino	725	*ANKS1A* genotype predicts cardiovascular events in patients with FH
Gallo	1624	A positive calcium score by CT predicts risk of future CVD
Mundal	5538	25% were hospitalized due to CVD in a 15-year period
Bogsrud	599	FH patients with high Lp(a) had twice the risk of CHD
Pavanello	350	Increased Lp(a) increased risk of previous CVD
SAFEHEART	2752	FH patients had more than 3-fold angina pectoris compared to controls
Mundal	4273	FH patients 25–49 had a higher excess risk of AMI both (women and men)
Svendsen	4871	FH patients have increased mortality and recurrent AMI after their first AMI
SAFEHEART	2752	FH patients had 3.1 fold AMI compared to controls

N = denotes number FH + patients.

LDL: Low-Density Lipoprotein.

IMT: intima media thickness.

When FH + patients was compared to unaffected relatives, the LDL receptor mutation type only partially contributed to the risk of future CVD (Umans-Eckenhausen et al., 2002).

When genotype-phenotype interactions were studied in FH + children the CVD risk in the parents was dependent on type of mutation (b).

In FH + patients, the presence of tendon xantomas was associated with increased risk of CVD (Civeira et al., 2005).

Small, dense LDL-particles in FH + patients were more prominent in patients with CVD (Soška et al., 2012)

Patients with severe FH + defined as LDL-C >8.0 mmol/L had increased risk of CVD with an adjusted hazard rate of 1.25 (Besseling et al., 2014) compared to those with lower LDL-C.

Using genetic risk scores in FH + patients, further enhanced prediction of CVD (Paquette et al., 2017). Also, recently, the same group has documented that rs17609940 variant of the *ANKS1A* gene is associated with increase in CVD events in FH + patients (Fantino et al., 2021).

Very recently, data from the prospective French and Spanish FH registries were combined, and 1624 patients with both FH+ and a coronary artery calcium score were included, and they established a high risk of future ACVD if the calcium score was above 100 (Gallo et al., 2021).

## Familial hypercholesterolemia-mutation and coronary artery disease

### Stable coronary artery disease

(Table 1) In a prospective registry study of FH + patients, 1411 patients were hospitalized for a CVD related diagnosis in a 15-year period, and ischemic heart disease was the most common diagnoses at admission reported in 90% of the hospitalizations (Mundal et al., 2016). Lipoprotein(a) is an important risk factor for ASCVD, so also in FH+; in FH patients with low lipoprotein(a), 7.8% had angina pectoris, while in the high lipoprotein(a) group, 16.7% had angina pectoris (Bogsrud et al., 2019). Similar results were also reported by Pavenello et al. from two cohorts of FH patients that previous episodes of CVD were associated with higher levels of lipoprotein(a) (Pavanello et al., 2019).

The prospective SAFEHEART registry study including FH + patients (average age 44, 46% males) compared to unaffected relatives (average age 40, 47% males) found a 3.4-fold increased prevalence of angina pectoris in the FH group (Pérez de Isla et al., 2016). This study also demonstrated that lipoprotein(a) is a risk factor for CVD in mutation positive FH patients (Alonso et al., 2014).

#### Acute coronary syndromes

In FH + patients without prior AMI, 99 incident AMI was observed during 2001–2009 in Norway, which is 3.58 AMI per thousand patients per year with an average age of 56.2 (13.4) at the time of the first event (Mundal et al., 2018). Importantly, the standardized incidence ratios (SIRs) with 95% CIs for AMI (95% CIs) were highest in the young age group 25–39 years; 7.5 (3.7–14.9) in men and 13.6 (5.1–36.2) in women, and decreased down to close to one at age 79–79 years (Mundal et al., 2018). This study population was later expanded and studied during 2001–2017 to identify 232 patients with an incident AMI which is 2.98 AMI per thousand patients per year in the expanded cohort (Svendsen et al., 2021). The SAFEHEART registry reported a 3.1-fold increased prevalence of AMI in the FH + group compared to controls (Pérez de Isla et al., 2016).

In 103 patients with ACS with a mean age of 55 with LDL-C> 4.1 mmol/L, 87% men, and offered these patients genetic testing and found that the prevalence of genetically confirmed FH was 9% in this group (Amor-Salamanca et al., 2017). This was also confirmed in a study where 130 young patients (<45 years) with LDL-C levels >4.0 mmol/L with acute MI, were offered genetic testing (Bogsrud et al., 2020). Of these, fifty-two patients were genetically tested and 11 was identified with a positive FH diagnosis, constituting a prevalence of 8.5%, in accordance with the results from Amor-Salamanca et al.

By means of cascade testing FH + patients were identified and offered statin therapy and compared to a matched control group (10:1). The median follow-up time was 21 years, and a coronary event (AMI, PCI, CABG) was found in 23% of the FH patients and in 4% of the control population (Kjærgaard et al., 2017).

FH + patients have increased risk of coronary artery disease, but an unanswered question is whether the excess risk of other types of ASCVD is increased to a similar degree? To compare the risk of various types of CVD it is an advantage to study incidence in the same cohort during the same period. We here present risk of various types of ASCVD studied in all FH + patients registered in Norway either at 31 Dec 2009 or at 31 April 2014 and discuss these data together with updated relevant literature in the field.

### Familial hypercholesterolemia and cerebrovascular disease

In 3166 FH + patients studied during more than 18 500 person years there was no increased risk of cerebrovascular disease or ischemic stroke (Hovland et al., 2019). A recent study confirmed the finding of no association between FH+ and stroke adjusted for cumulative statin exposure in the FH + group (Svendsen et al., 2022). Similarly, in the prospective SAFEHEART registry of FH + patients there was no increase in any atherosclerotic cerebrovascular disease (Pérez de Isla et al., 2016). Kjærgård et al. studying 118 FH + patients compared to 102 non-mutation carrying relatives with a median follow-up time of 21 years found the occurrence of stroke in 6% of the subjects in the FH group compared to 5% in the control group (Kjærgaard et al., 2017). In the Copenhagen General Population Study the cumulative incidence of ischemic stroke was similar in 185 FH + individuals compared to 106227 individuals with no FH mutation (Beheshti et al., 2018). Also, we have not found any association between FH+ and dementia (Mundal et al., 2022).

A Mendelian randomization study has demonstrated that people with lifelong low levels of PCSK9 and LDL-C had lower risk of coronary heart disease but with no effect on ischemic stroke (Hopewell et al., 2018). Taken together, the pathophysiological relation between LDL-C and ischemic strokes in FH + patients appears to be unclear at present.

## Familial hypercholesterolemia and peripheral artery disease

In 3162 FH + patients compared to the general Norwegian population, we found that the risk of peripheral artery disease was nearly tripled in the FH group compared to controls in both women and men (Mundal et al., 2020).

Pereira et al. also demonstrated an increased risk of peripheral artery disease in FH (90% FH+) patients with a mean age of 51 years compared to normolipidemic persons, with a prevalence of peripheral artery disease assessed by ankle-brachial index of 17.3 vs 2.3% respectively, the patients were older in the FH group, and there was more diabetes in the FH group, however there was a higher prevalence of smokers in the control group (Pereira et al., 2015).

In a study investigating presence of comorbidities at time of death among 79 mainly FH + patients, 39% of the patients had peripheral artery disease (diseases of arteries, arterioles and capillaries) at time of death at mean 60 years of age (Krogh et al., 2016).

The SAFEHEART registry with 2752 FH + patients with a mean age of 44 years upon enrollment was compared to 993 unaffected relatives showed 39 cases of any peripheral artery disease in the FH group compared to two cases in the control group, and 14 cases of peripheral artery revascularization in the FH group, compared to none in the control group (Pérez de Isla et al., 2016).

Indeed, these studies suggest that the risk of peripheral artery disease is increased in FH, and accordingly, increased LDL-C burden during life should be considered a risk factor for this kind of ASCVD.

## Familial hypercholesterolemia and aortic disease

We have recently demonstrated an increased risk of aortic disease including abdominal aortic aneurisms in FH + men, but not in women (Mundal et al., 2021). LDL-C is thought to be a risk factor for aortic aneurisms, and a Mendelian randomized trial suggests that predicted LDL-C increase the risk of aortic aneurisms (Allara et al., 2019). In 79 patients mainly mutation positive FH patients, 14% of the patients had presence of aortic aneurysm at time of death at mean 60 years of age (Krogh et al., 2016).

### Cardiovascular disease mortality

In a cohort of 4688 FH + patients, risk of CVD death was increased compared to the rest of the Norwegian population in all age groups below 70 years (Mundal et al., 2014). These data were later extended and in 5518 FH + patients in whom standardized mortality ratios was significantly higher in FH patients compared to matched controls, particularly in the young, and for those aged 20–39 years, the risk of cardiovascular disease death during hospitalization was increased 12-fold (Mundal et al., 2017). In another cohort, 93% of FH + had established CVD at the time of death, and 69% of them had experienced myocardial infarction (Krogh et al., 2016).

## Other cardiovascular diseases

### Heart failure and atrial fibrillation

Increased risk of heart failure was reported in 4273 Norwegian FH + patients compared to the general Norwegian population, and the highest excess risk was observed in the younger age group 25–49 years, and most of the heart failure patients had previous coronary artery disease (Hovland et al., 2017). We also studied the risk of atrial fibrillation in FH patients and found a doubling of atrial fibrillation and atrial flutter among the FH patients compared to the controls (Hovland et al., 2017). Krogh et al. showed that 19% of mainly FH + patients had presence of atrial fibrillation whereas 35% had heart failure at time of death at mean 60 years of age (Krogh et al., 2016).

Coronary heart diseases including myocardial infarction is a common disease in patients with familial hypercholesterolemia and therefore the increased risk of heart failure and atrial fibrillation could be expected as coronary heart disease is a common risk factor for both.

### Aortic valve stenosis

LDL-C seems to be important in the early phase of aortic valve stenosis build up (Peeters et al., 2018), however, LDL-C lowering therapy has been futile in halting its progress (Zhao et al., 2016). We found 55 cases of a first-time diagnosis of aortic valve stenosis in 3161 FH + patients in a prospective registry from 2001 during 2009 which constitutes 1.93 incident cases per thousand patients per year. Compared to the Norwegian population and adjusted for age and sex, SIR was 7.9 (95% CI, 6.1–10.4) (Mundal et al., 2019). Perez de Isla et al. have confirmed our findings in the SAFEHEART prospective cohort of 3712 FH + patients compared to non-affected relatives in which the odds ratio (95% CI) for having an aortic valve replacement was 5.71 (1.8–18.4) after a mean follow-up of 7.5 years (Pérez de Isla et al., 2021).

In a study of homozygous FH, Alonso et al. showed that patients with null mutations had higher levels of LDL-C and more aortic valve stenosis than patients with a receptor defective mutation, indicating that the level of LDL-C is important for progression of this disease (Alonso et al., 2016).

The association of increased risk of aortic valve stenosis in FH+ is quite clear despite those previous studies of LDL-C lowering in aortic valve stenosis being negative. Most of the studies on aortic valve stenosis have been performed relatively late in the disease, and early intervention is less studied. However, this could be further explored in FH patients. New treatment principles including PCSK9-inhibitors, inclisiran and evinacumab facilitate LDL-C- lowering in FH patients to even lower levels than we have achieved earlier and might be tested out with respect to aortic valve stenosis progression. Data suggest that high lipoprotein(a) values in particular increase the risk of aortic stenosis (Mach et al., 2019). This might be particularly important for FH + patients, because a 2-3 doubling of an already high risk will constitute a significant difference in the absolute risk. Thus, specific lowering of lipoprotein(a) using new antisense oligonucleotides treatment in the future may become a particularly important option for FH + patients with high lipoprotein(a) levels, when such drugs are eventually approved.

### The spectrum of atherosclerotic cardiovascular disease in familial hypercholesterolemia

**FIGURE 1:**
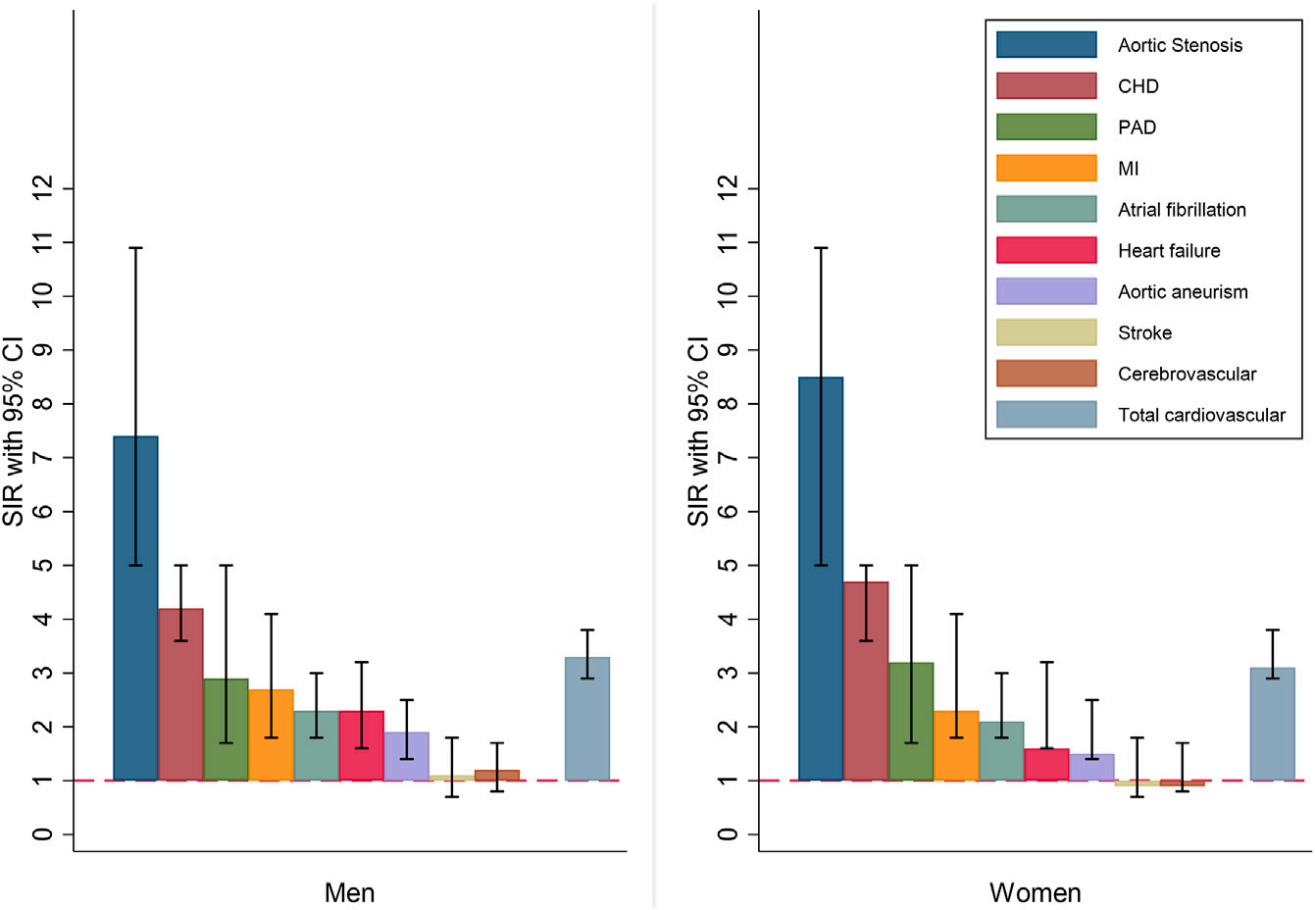
Standardized incidence ratios (SIRs) for different types of cardiovascular disease in men and women. CHD: Coronary Heart disease; PAD: Peripheral artery disease; MI: Myocardial infarction.

Regarding the increased risk of different kinds of ASCVD in patients with FH + there seems to be a wide range in the increased risk induction of the different diseases (Figure 1 and Supplementary Figure S1 and Supplementary Tables S1–S3). As an example we found that the excess risk of aortic stenosis and coronary heart disease is quite high (Mundal et al., 2016; Mundal et al., 2019), whereas there is no excess risk in risk of cerebrovascular disease and stroke (Hovland et al., 2019).

FH + patients are at a markedly increased risk of a broad range of ASCVD. AMI is the most common in absolute numbers, however, aortic valve stenosis is by far associated with the highest excess risk. Per thousand patients, we observed 3.6 incident AMI per year compared to 1.9 incident aortic valve stenosis, however, SIR for incident AMI was 2.3 compared to 7.9 for incident aortic valve stenosis. Further, occurrence of ischemic stroke seems not to be associated with increased risk in FH+.

It should be mentioned that most of the data on FH + are collected in Caucasian persons, and it remains to be studied whether this applies to all races.

## Familial hypercholesterolemia–implications for the clinician

Clinicians should be aware of the excess risk of almost all kind of ASCVD in FH+, and the neutral risk of stroke need to be studied further in FH + patients. Clinicians should suspect an FH diagnosis in patients with high levels of LDL-C (>5.0 mmol/L in adults), and a family history of premature ASCVD. Fewer FH patients have xanthoma now compared to earlier times, as many are treated with lipid lowering drugs, however without knowing of the FH diagnosis. Further, as the number of treated FH patients increases, there will be less ASCVD in the parent generation which weakens the accuracy of the clinical scoring systems for FH. In emergency rooms and coronary care units, FH patients may present with early onset coronary artery disease, and an FH diagnosis should be suspected in young patients with high LDL-C. In suspected FH, genetic counselling and testing should then be offered to establish treatment and to perform cascade testing if a mutation is demonstrated. Early detection and correct treatment are paramount in reducing the risk of ASCVD among FH + patients.

## References

[B1] Allara E. Andres E. Mata N. Fuentes-Jimenez F. Badimon L. Lopez-Miranda J. (2019). Lipoprotein(a) levels in familial hypercholesterolemia: An important predictor of cardiovascular disease independent of the type of LDL receptor mutation. J. Am. Coll. Cardiol. 63, 1982–1989. 10.1016/j.jacc.2014.01.063 24632281

[B2] Alonso R. Diaz-Diaz J. L. Arrieta F. Fuentes-Jimenez F. de Andres R. Saenz P. (2016). Clinical and molecular characteristics of homozygous familial hypercholesterolemia patients: Insights from SAFEHEART registry. J. Clin. Lipidol. 10, 953–961. 10.1016/j.jacl.2016.04.006 27578128

[B3] Amor-Salamanca A. Castillo S. Gonzalez-Vioque E. Dominguez F. Quintana L. Lluis-Ganella C. (2017). Genetically confirmed familial hypercholesterolemia in patients with acute coronary syndrome. J. Am. Coll. Cardiol. 70, 1732–1740. 10.1016/j.jacc.2017.08.009 28958330

[B4] Arnesen K. E. Phung A. V. Randsborg K. Mork I. Thorvall M. Langslet G. (2020). Risk of recurrent coronary events in patients with familial hypercholesterolemia; A 10-years prospective study. Front. Pharmacol 11, 560958. 10.3389/fphar.2020.560958 33737874 PMC7961401

[B5] Attipoe-Dorcoo S. Kindt I. Hof M. Kastelein J. J. P. Hutten B. A. Hovingh G. K. (2021). Characteristics and trends of PCSK9 inhibitor prescription fills in the United States. J. Clin. Lipidol. 10.1016/j.jacl.2021.01.010 33589405

[B6] Beheshti S. Madsen C. M. Varbo A. Benn M. Nordestgaard B. G. (2018). Relationship of familial hypercholesterolemia and high low-density lipoprotein cholesterol to ischemic stroke: Copenhagen general population study. Circulation 138, 578–589. 10.1161/CIRCULATIONAHA.118.033470 29593013

[B7] Besseling J. Kindt I. Hof M. Kastelein J. J. P. Hutten B. A. Hovingh G. K. (2014). Severe heterozygous familial hypercholesterolemia and risk for cardiovascular disease: A study of a cohort of 14, 000 mutation carriers. Atherosclerosis 233, 219–223. 10.1016/j.atherosclerosis.2013.12.020 24529147

[B8] Betteridge J. D. Græsdal A. Johansen D. Langslet G. Hovland A. Arnesen K. E. (2000). Lipids and vascular disease: Current issues. Taylor Francis.

[B9] Bogsrud M. P. Græsdal A. Johansen D. Langslet G. Hovland A. Arnesen K. E. (2019). LDL-cholesterol goal achievement, cardiovascular disease, and attributed risk of Lp(a) in a large cohort of predominantly genetically verified familial hypercholesterolemia. J. Clin. Lipidol. 13, 279–286. 10.1016/j.jacl.2019.01.010 30910667

[B10] Bogsrud M. P. Oyri L. K. L. Halvorsen S. Atar D. Leren T. P. Holven K. B. (2020). Prevalence of genetically verified familial hypercholesterolemia among young (<45 years) Norwegian patients hospitalized with acute myocardial infarction. J. Clin. Lipidol. 14, 339–345. 10.1016/j.jacl.2020.04.002 32418822

[B11] Brorholt-Petersen J. U. Jensen H. K. Jensen J. M. Refsgaard J. ChrisTiansen T. Hansen L. B. (2002). LDL receptor mutation genotype and vascular disease phenotype in heterozygous familial hypercholesterolaemia. Clin. Genet. 61, 408–415. 10.1034/j.1399-0004.2002.610603.x 12121347

[B12] Civeira F. Castillo S. Alonso R. Merino-Ibarra E. Cenarro A. Artied M. (2005). Tendon xanthomas in familial hypercholesterolemia are associated with cardiovascular risk independently of the low-density lipoprotein receptor gene mutation. Arterioscler. Thromb. Vasc. Biol. 25, 1960–1965. 10.1161/01.ATV.0000177811.14176.2b 16020744

[B13] Domanski M. J. Tian X. Wu C. O. Reis J. P. Dey A. K. Gu Y. (2020). Time course of LDL cholesterol exposure and cardiovascular disease event risk. J. Am. Coll. Cardiol. 76, 1507–1516. 10.1016/j.jacc.2020.07.059 32972526

[B14] Fantino M. Malik R. Valdes-Marquez E. Worrall B. B. Collins R. (2021). ANKS1A genotype predicts cardiovascular events in patients with familial hypercholesterolemia,. J. Clin. Lipidol. 10.1016/j.jacl.2021.05.006 34130940

[B15] Gallo A. Perez de Isla L. Charriere S. Vimont A. Alonso R. Muniz-Grijalvo O. (2021). The added value of coronary calcium score in predicting cardiovascular events in familial hypercholesterolemia. JACC. Cardiovasc. Imaging 14, 2414–2424. 10.1016/j.jcmg.2021.06.011 34274263

[B16] Group S. S. C. O. B. O. T. S. B. R. (1991). Risk of fatal coronary heart disease in familial hypercholesterolaemia. Scientific Steering Committee on behalf of the Simon Broome Register Group. BMJ 303, 893–896. 10.1136/bmj.303.6807.893 1933004 PMC1671226

[B17] Hopewell J. C. Malik R. Valdés-Márquez E. Worrall B. B. Collins R. (2018). Differential effects of PCSK9 variants on risk of coronary disease and ischaemic stroke. Eur. Heart J. 39, 354–359. 10.1093/eurheartj/ehx373 29020353 PMC5837489

[B18] Hovland A. Mundal L. J. Igland J. Veierod M. B. Holven K. B. Bogsrud M. P. (2017). Increased risk of heart failure and atrial fibrillation in heterozygous familial hypercholesterolemia. Atherosclerosis 266, 69–73. 10.1016/j.atherosclerosis.2017.09.027 28992466

[B19] Hovland A. Mundal L. J. Igland J. Veierod M. B. Holven K. B. Bogsrud M. P. (2019). Risk of ischemic stroke and total cerebrovascular disease in familial hypercholesterolemia. Stroke 50, 172–174. 10.1161/STROKEAHA.118.023456 30580708

[B20] Hu P. Dharmayat K. I. Stevens C. A. T. Sharabiani M. T. A. Jones R. S. Watts G. F. (2020). Prevalence of familial hypercholesterolemia among the general population and patients with atherosclerotic cardiovascular disease: A systematic review and meta-analysis. Circulation 141, 1742–1759. 10.1161/CIRCULATIONAHA.119.044795 32468833

[B21] Khera A. V. Hegele R. A. (2020). What is familial hypercholesterolemia, and Why Does it matter. Circulation 141, 1760–1763. 10.1161/CIRCULATIONAHA.120.046961 32479201 PMC7299543

[B22] Kjærgaard K. A. Wiegman A. Rodenburg J. Defesche J. C. Kastelein J. J. P. Sijbrands E. J. G. (2017). Long-term cardiovascular risk in heterozygous familial hypercholesterolemia relatives identified by cascade screening. J. Am. Heart Assoc. 6, e005435. 10.1161/JAHA.116.005435 28652386 PMC5669167

[B23] Koeijvoets K. C. M. C. Wiegman A. Rodenburg J. Defesche J. C. Kastelein J. J. P. Sijbrands E. J. G., (2005). Effect of low-density lipoprotein receptor mutation on lipoproteins and cardiovascular disease risk: A parent-offspring study. Atherosclerosis 180, 93–99. 10.1016/j.atherosclerosis.2004.10.042 15823280

[B24] Krogh H. W. Mundal L. Holven K. B. Retterstol K. (2016). Patients with familial hypercholesterolaemia are characterized by presence of cardiovascular disease at the time of death. Eur. Heart J. 37, 1398–1405. 10.1093/eurheartj/ehv602 26586781

[B25] Lee S. Akioyamen L. E. Aljenedil S. Rivière J. B. Ruel I. Genest J., (2019). Genetic testing for familial hypercholesterolemia: Impact on diagnosis, treatment and cardiovascular risk. J. Prev. 10.1177/2047487319829746 30755017

[B26] Mach F. Baigent C. Catapano A. L. Koskinas K. C. Casula M. Badimon L. (2019). 2019 ESC/EAS guidelines for the management of dyslipidaemias: Lipid modification to reduce cardiovascular risk. Eur. Heart J. 41, 111–188. 10.1093/eurheartj/ehz455 31504418

[B27] MedPedProgram U. S. (2005). www.medped.org.medped.org.

[B28] Mundal L. Igland J. Ose L. Holven K. B. Veierod M. B. Leren T. P. (2017). Cardiovascular disease mortality in patients with genetically verified familial hypercholesterolemia in Norway during 1992-2013. Eur. J. Prev. Cardiol. 24, 137–144. 10.1177/2047487316676135 27794106

[B29] Mundal L. J. Hovland A. Igland J. Veierød M. B. Holven K. B. Bogsrud M. P. (2019). Association of low-density lipoprotein cholesterol with risk of aortic valve stenosis in familial hypercholesterolemia. JAMA Cardiol. 4, 1156–1159. 10.1001/jamacardio.2019.3903 31617858 PMC6802245

[B30] Mundal L. J. Igland J. Leren T. P. Retterstol K. (2021). Excess aortic pathology risk in patients with genetically verified familial hypercholesterolaemia: A prospective Norwegian registry study. Eur. J. Vasc. Endovascular Surg. 61, 712–713. 10.1016/j.ejvs.2020.12.019 33485759

[B31] Mundal L. J. Hovland A. Igland J. Veierod M. B. Holven K. B. Bogsrud M. P. (2020). Increased risk of peripheral artery disease in persons with familial hypercholesterolaemia: A prospective registry study. Eur. J. Prev. Cardiol. 28, e11–e13. 10.1093/eurjpc/zwaa024 33623989

[B32] Mundal L. J. Igland J. Svendsen K. Holven K. B. Leren T. P. Retterstol K. (2022). Association of familial hypercholesterolemia and statin use with risk of dementia in Norway. JAMA Netw. Open 5, e227715. 10.1001/jamanetworkopen.2022.7715 35438756 PMC9020214

[B33] Mundal L. J. Igland J. Veierod M. B. Holven K. B. Ose L. Selmer R. M. (2018). Impact of age on excess risk of coronary heart disease in patients with familial hypercholesterolaemia. Heart 104, 1600–1607. 10.1136/heartjnl-2017-312706 29622598 PMC6161660

[B34] Mundal L. Sarancic M. Ose L. Iversen P. O. Borgan J. K. Veierod M. B. (2014). Mortality among patients with familial hypercholesterolemia: A registry-based study in Norway, 1992-2010. J. Am. Heart Assoc. 3, e001236. 10.1161/JAHA.114.001236 25468658 PMC4338710

[B35] Mundal L. Veierod M. B. Halvorsen T. Holven K. B. Ose L. Iversen P. O. (2016). Cardiovascular disease in patients with genotyped familial hypercholesterolemia in Norway during 1994-2009, a registry study. Eur. J. Prev. Cardiol. 23, 1962–1969. 10.1177/2047487316666371 27558979

[B36] Paquette M. Chong M. Theriault S. Dufour R. Pare G. Baass A. (2017). Polygenic risk score predicts prevalence of cardiovascular disease in patients with familial hypercholesterolemia. J. Clin. Lipidol. 11, 725–732. 10.1016/j.jacl.2017.03.019 28456682

[B37] Pavanello C. Pirazzi C. Bjorkman K. Sandstedt J. Tarlarini C. Mosca L. (2019). Individuals with familial hypercholesterolemia and cardiovascular events have higher circulating Lp(a) levels. J. Clin. Lipidol. 13, 778–787. 10.1016/j.jacl.2019.06.011 31371270

[B38] Peeters F. E. C. M. Meex S. J. R. Dweck M. R. Aikawa E. Crijns H. J. G. M. Schurgers L. J. (2018). Calcific aortic valve stenosis: Hard disease in the heart: A biomolecular approach towards diagnosis and treatment. Eur. Heart J. 39, 2618–2624. 10.1093/eurheartj/ehx653 29136138 PMC6055545

[B39] Pereira C. Miname M. H. Makdisse M. R. P. Watanabe C. Pesaro A. E. Jannes C. E. (2015). Peripheral arterial disease in heterozygous familial hypercholesterolemia. Atherosclerosis 242, 174–178. 10.1016/j.atherosclerosis.2015.07.022 26201001

[B40] Pérez de Isla L. Alonso R. Mata N. Saltijeral A. Muniz O. Rubio-Marin P. (2016). Coronary heart disease, peripheral arterial disease, and stroke in familial hypercholesterolaemia: Insights from the SAFEHEART registry (Spanish familial hypercholesterolaemia cohort study). Arterioscler. Thromb. Vasc. Biol. 36, 2004–2010. 10.1161/ATVBAHA.116.307514 27444203

[B41] Perez de Isla L. Rosenson R. S. Reeskamp L. F. Hovingh G. K. Kastelein J. J. Rubba P. (2021)). Lipoprotein(a), LDL-cholesterol, and hypertension: Predictors of the need for aortic valve replacement in familial hypercholesterolaemia. Eur. Heart J. 42, 2201–2211. 10.1093/eurheartj/ehaa1066 33437997

[B42] Raal F. J. Rosenson R. S. Reeskamp L. F. Hovingh G. K. Kastelein J. J. P. Rubba P. (2020). Evinacumab for homozygous familial hypercholesterolemia. N. Engl. J. Med. Overseas. Ed. 383, 711–720. 10.1056/nejmoa2004215 32813947

[B43] Soška V. Jarkovsky J. Ravcukova B. Tichy L. Fajkusova L. Freiberger T. (2012). The logarithm of the triglyceride/HDL-cholesterol ratio is related to the history of cardiovascular disease in patients with familial hypercholesterolemia. Clin. Biochem. 45, 96–100. 10.1016/j.clinbiochem.2011.11.001 22119890

[B44] Svendsen K. Krogh H. W. Igland J. Tell G. S. Mundal L. J. Holven K. B. (2021). 2.5-fold increased risk of recurrent acute myocardial infarction with familial hypercholesterolemia. Atherosclerosis 319, 28–34. 10.1016/j.atherosclerosis.2020.12.019 33465659

[B45] Svendsen K. Olsen T. Vinknes K. J. Mundal L. J. Holven K. B. Bogsrud M. P. (2022). Risk of stroke in genetically verified familial hypercholesterolemia: A prospective matched cohort study. Atherosclerosis 358, 34–40. 10.1016/j.atherosclerosis.2022.08.015 36084445

[B46] Trinder M. Li X. DeCastro M. L. Cermakova L. Sadananda S. Jackson L. M. (2019). Risk of premature atherosclerotic disease in patients with monogenic versus polygenic familial hypercholesterolemia. J. Am. Coll. Cardiol. 74, 512–522. 10.1016/j.jacc.2019.05.043 31345425

[B47] Umans-Eckenhausen M. A. Sijbrands E. J. G. Kastelein J. J. P. Defesche J. C. (2002). Low-density lipoprotein receptor gene mutations and cardiovascular risk in a large genetic cascade screening population. Circulation 106, 3031–3036. 10.1161/01.cir.0000041253.61683.08 12473547

[B48] Virani S. S. Alonso A. Benjamin E. J. Bittencourt M. S. Callaway C. W. Carson A. P. (2021). Heart disease and stroke statistics-2020 update: A report from the American heart association. Circulation 141, e139–e596. 10.1161/CIR.0000000000000757 31992061

[B49] Zhao Y. Nicoll R. He Y. H. Henein M. Y. (2016). The effect of statins on valve function and calcification in aortic stenosis: A meta-analysis. Atherosclerosis 246, 318–324. 10.1016/j.atherosclerosis.2016.01.023 26828749

