# Supplementary material for: The risk of various types of cardiovascular diseases in mutation positive familial hypercholesterolemia; a review
Source: Front Genet. 2022 Dec 6;13:1072108. doi: 10.3389/fgene.2022.1072108 (PMC9763610; doi:10.3389/fgene.2022.1072108)
Supplement: Supplementary file 1 [file DataSheet1.PDF]

Table 1: Standardized incidence ratio (SIR) for different subgroups of cardiovascular disease, age- and sex-standardized according to the distribution of the general Norwegian population

	Person-years of follow-up in 1000	Incident cases	Age at time of incident event, mean (sd)	Age at time of incident event, mean (95% CI)	Crude incidence rate per 1000 person years (95% CI)	Expected number of cases	SIR (95% CI)
Total cardiocascular disease (CVD)*	144.4	344	53.7 (13.0)	53.7 (52.3-55.1)	23.8 (21.4-26.5)	107.6	3.2 (2.9-3.6)
Aortic stenosis	18.26	53	65.1 (10.2)	65.1 (62.3-67.9)	2.9 (2.2-3.8)	6.7	7.9 (6.1-10.4)
CHD	15.45	253	55.1 (13.0)	55.1 (53.5-56.7)	16.4 (14.5-18.5)	57.2	4.4 (3.9-5.0)
PAD	18.35	40	58.5 (11.2)	58.4 (54.8-61.9)	18.3 (1.6-3.0)	13.7	2.9 (2.1-4.0)
MI	17.72	28	56.2 (13.4)	56.2 (53.5-58.8)	5.6 (4.6-6.8)	42.7	2.3 (1.9-2.8)
Atrial fibrillation	18.2	77	65.2 (12.8)	65.2 (62.3-68.1)	4.2 (3.4-5.3)	39.4	2.0 (1.6-2.4)
Heart failure	18.3	54	62.2 (12.7)	62.2 (58.8-65.7)	2.9 (2.3-3.8)	27.6	2.0 (1.5-2.6)
Aortic aneurism	18.5	17	62.2 (6.9)	62.2 (58.6-65.7)	0.9 (0.6-1.5)	6.8	2.5 (1.6-4.0)
Stroke	18.4	32	59.9 (14.4)	59.9 (54.7-65.1)	1.7 (1.2-2.5)	31.9	1.0 (0.7-1.4)
Cerebrovascular	18.4	46	59.5 (13.2)	59.5 (55.6-63.5)	2.5 (1.9-3.4)	44.8	1.0 (0.8-1.4)

*ICD10 I00-I99

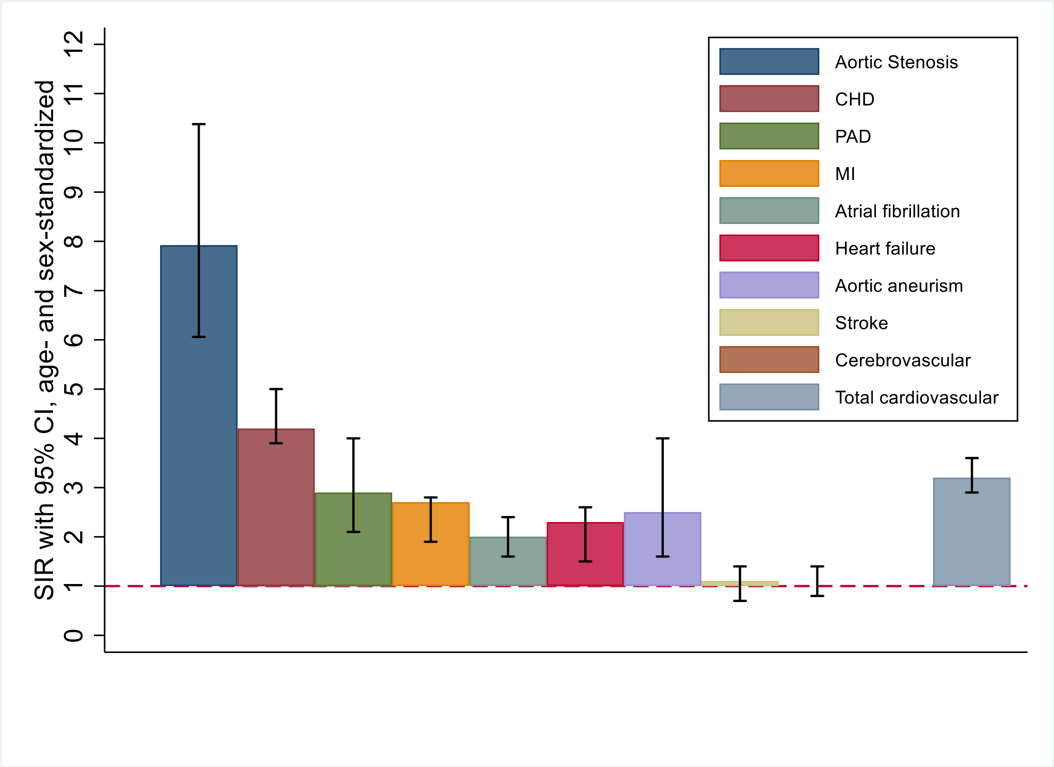

Figure 1: Men and women combined, age- and sex-standardized SIR.

Table 2: Age at first event, women

	Incident cases	Age at time of incident event, mean (sd)	Age at time of incident event, mean (95% CI)
Total cardiocascular disease (CVD)*	158	55.9 (13.7)	55.9 (53.7-58.0)
Aortic stenosis	27	69.0 (10.2)	69.0 (65.0-73.0)
CHD	111	57.7 (13.4)	57.7 (55.2-60.2)
PAD	19	59.9 (12.3)	59.9 (54.0-65.8)
MI	34	60.1 (13.7)	60.1 (55.3-64.9)
Atrial fibrillation	33	72.2 (12.0)	72.2 (68.0-76.5)
Heart failure	20	68.8 (13.3)	68.8 (62.5-75.0)
Aortic aneurism	3	65.7 (4.0)	65.7 (55.6-75.7)
Stroke	13	67.9 (13.4)	67.9 (59.8-76.0)
Cerebrovascular	19	63.7 (12.7)	63.7 (57.6-69.9)

Table 3: Age at first event, men

	Incident cases	Age at time of incident event, mean (sd)	Age at time of incident event, mean (95% CI)
Total cardiocascular disease (CVD)*	186	51.8 (12.1)	51.8 (50.1-53.6)
Aortic stenosis	26	61.0 (8.9)	61.0 (57.4-64.6)
CHD	142	53.1 (12.4)	53.1 (51.0-55.1)
PAD	21	57.0 (10.3)	57.0 (52.3-61.6)
MI	65	54.1 (12.9)	54.1 (50.9-57.3)
Atrial fibrillation	44	59.9 (10.8)	59.9 (56.6-63.2)
Heart failure	34	58.4 (10.8)	58.4 (64.6-62.2)
Aortic aneurism	14	61.4 (6.9)	61.4 (57.2-65.6)
Stroke	19	54.4 (12.6)	54.4 (48.3-60.5)
Cerebrovascular	27	56.6 (12.0)	56.6 (51.4-61.7)
